# Non-thermal effects of radiofrequency electromagnetic fields

**DOI:** 10.1038/s41598-020-69561-3

**Published:** 2020-08-10

**Authors:** Peter Wust, Benedikt Kortüm, Ulf Strauss, Jacek Nadobny, Sebastian Zschaeck, Marcus Beck, Ulrike Stein, Pirus Ghadjar

**Affiliations:** 1 Department of Radiation Oncology, Charité–Universitätsmedizin Berlin, Corporate Member of Freie Universität Berlin, Humboldt-Universität zu Berlin, and Berlin Institute of Health, Augustenburger Platz 1, 13353 Berlin, Germany; 2 Experimental and Clinical Research Center, Charité–Universitätsmedizin Berlin and Max-Delbrück-Centrum (MDC), Robert-Rössle-Str. 10, 13092 Berlin, Germany; 3 Institute of Cellbiology and Neurobiology, Charité–Universitätsmedizin Berlin, Corporate Member of Freie Universität Berlin, Humboldt-Universität zu Berlin, and Berlin Institute of Health, Charitéplatz 1, 10117 Berlin, Germany; 4 Berlin Institute of Health (BIH), Anna-Louisa-Karsch Str. 2, 10178 Berlin, Germany; 5 German Cancer Consortium (DKTK), Im Neuenheimer Feld 280, 69120 Heidelberg, Germany

**Keywords:** Biophysics, Biotechnology, Cancer

## Abstract

We explored the non-thermal effects of radiofrequency (RF) electromagnetic fields and established a theoretical framework to elucidate their electrophysiological mechanisms. In experiments, we used a preclinical treatment device to treat the human colon cancer cell lines HT-29 and SW480 with either water bath heating (WB-HT) or 13.56 MHz RF hyperthermia (RF-HT) at 42 °C for 60 min and analyzed the proliferation and clonogenicity. We elaborated an electrical model for cell membranes and ion channels and estimated the resulting ion fluxes. The results showed that, for both cell lines, using RF-HT significantly reduced proliferation and clonogenicity compared to WB-HT. According to our model, the RF electric field component was rectified and smoothed in the direction of the channel, which resulted in a DC voltage of ~ 1 µV. This may induce ion fluxes that can potentially cause relevant disequilibrium of most ions. Therefore, RF-HT creates additional non-thermal effects in association with significant ion fluxes. Increasing the understanding of these effects can help improve cancer therapy.

## Introduction

Electromagnetic fields (EMF) are generally believed to have no relevant non-thermal effects on cells, tissues, and living organisms^1,2^. Only EMF with an excessive strength of > 1.000 kV/m exhibits non-thermal membrane effects such as electroporation^3^ or bactericidal microwave exposure^4^. Recently, non-thermal effects have been clinically exploited with the tumor-treating field method^5,6^, which applies an EMF at radio frequencies (RF) of 100–300 kHz with a moderate strength of 100–150 V/m. The scientific community considers the risk of such moderate-strength RF-EMF to be negligible, at least with respect to potential hazards caused by powerlines or mobile phones^7–11^. Nevertheless, some unresolved observations remain^12,13^, and further research is still recommended^14^. However, further specific investigations are only rarely performed because the mechanisms of non-thermal effects remain unknown^15^.

Oncologists have applied RF technology with similar EMF levels of 200 V/m to cancer treatment using either capacitive (8–30 MHz) or radiative (70–120 MHz) techniques^16^. The temperature increase is regarded as the major working mechanism^17^. Preclinical data for water bath hyperthermia (WB-HT) have indicated that a temperature of greater than 42 °C is required^18^. However, this is rarely achieved in clinical practice, and a temperature achieved in 90% of the target (T_90_) of only 39.5–40.5 °C has been correlated with effectiveness^19^. Meanwhile, a series of positive randomized trials has shown that RF hyperthermia (RF-HT) improves the effectiveness of radiotherapy or radio-chemotherapy for cervical cancer^20–22^. In contrast, extreme whole-body hyperthermia, as the clinical counterpart of WB-HT, using temperatures of ≥ 42 °C has proved to be less effective leading to disappointing results in cancer therapy^23^. This indicates that non-thermal effects of RF-EMF do exist.

Preclinical studies with animal tumors and cell suspensions^24–26^ have suggested that RF-HT at 13.56 MHz is considerably more effective than WB-HT or infrared heating at the same temperature. For instance, maintaining RF-HT at 13.56 MHz at 42 °C for 60 min has approximately the same cytotoxic effect as WB-HT maintained at 44 °C for 60 min.

However, pure sinusoidal RF at 13.56 MHz either with or without additional amplitude modulation of several kHz has not been distinguished rigorously in the literature^24–26^.

Differences between homogeneous WB-HT and RF-HT are often attributed to hotspots that cannot be discovered with conventional thermometry. We recently demonstrated that any kind of microscopic hotspot (i.e., nanoheating or point heating) would require excessive and unrealistic specific absorption rate (SAR) peaks (e.g., > 10,000 W/kg for millimeter-size spheres) and cause macroscopic temperature elevations^27^.

Non-thermal effects most likely occur at cell membranes, which have widely explored electrochemical behavior^28,29^. In the case of RF-EMF > 1 MHz (as used in clinical RF-HT), energy is transferred to the tissue by ionic and dielectric dissipation^30^, and the translational shifts of ions are considered too small (< 0.1 nm) to cause any relevant ion fluxes through the cell membrane. A previous theoretical analysis asserted that RF-EMF as high as 200 kV/m is required to excite cell membranes^31^.

To the best of our knowledge, research on the possible electrophysiological effects of RF is not available. In this study, therefore, we aimed to explore the non-thermal effects of RF-HT and establish a theoretical framework to discover potential working mechanisms, with an emphasis on specific electrophysiological membrane effects.

### Results

#### Experimental evidence for the non-thermal effects of RF-EMF

**Figure 1:**
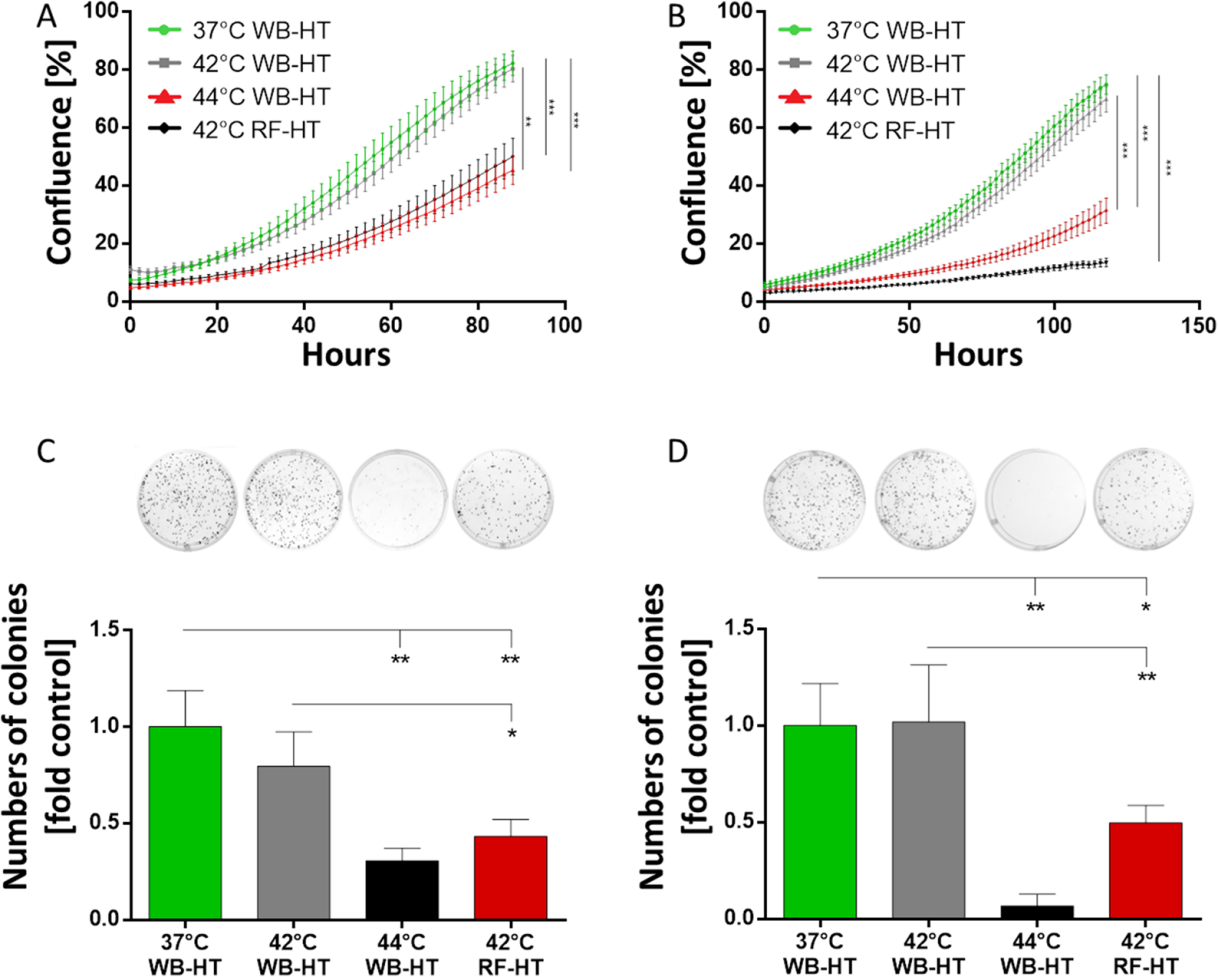
Radiofrequency hyperthermia (RF-HT) doubled the antiproliferative and anticlonogenic effects of conventional water bath hyperthermia (WB-HT) at 42 °C on colorectal cancer cells. For both the HT-29 (**A**, **C**) and SW480 cells (**B**, **D**), WB-HT at 42 °C (gray) did not significantly affect proliferation compared with WB-HT at 37 °C (green). RF-HT at 42 °C (red) drastically inhibited cell proliferation to an extent comparable to that of WB-HT at 44 °C (**A**, **B**) and also significantly reduced the numbers of clones detectable after 10 days for HT-29 (**C**) and SW480 cells (**D**).

As shown in Fig. 1, our experiments on the HT-29 and SW480 cell suspensions indicated that RF-HT reduced proliferation and clonogenicity twice as much as WB-HT at the same temperature of 42 °C. For both cell lines, proliferation and clonogenicity were significantly reduced after RF-HT compared with WB-HT alone. The proliferation of HT-29 cells decreased significantly after RF-HT at 42 °C compared with WB-HT at 37 and 42 °C (both *p* < 0.0001) (Fig. 1A). The proliferation of SW480 cells was similarly affected and reduced more by RF-HT at 42 °C than WB-HT at 37 and 42 °C (both *p* < 0.0001) (Fig. 1B). In the clonogenicity assays, RF-HT at 42 °C significantly reduced the number of colonies of HT-29 cells compared with WB-HT at 37 °C (*p* = 0.005) and 42 °C (*p* = 0.04) (Fig. 1C). The clonogenicity of the SW480 cells was similarly reduced by RF-HT at 42 °C compared with WB-HT at 37 °C (*p* = 0.05) and 42 °C (*p* = 0.04) (Fig. 1D).

#### Electrical model of the cell membrane and ion channels

We propose that ion channels act as half-wave rectifiers for the E-field component (i.e., voltage) across the membrane. Thus, the sinusoidal RF-EMF perpendicular to the membrane is converted into single positive half-waves. The capacitance (*C*_*X*_) of the channel of ion X embedded in the membrane and resistor of the channel (*R*_*X*_ = 1/*G*_*X*_), both in series with the rectifier, smoothen the half-waves. Finally, a DC voltage is superimposed with an RF ripple along this channel in the forward direction. For the given SAR of 25 W/kg (i.e., *E* = 200 V/m), we achieved a DC voltage of 1 µV at the channel. Generally, the smoothing effect increased with frequency RF and the time constant RC. When we tested the smoothing condition of the equivalent circuit diagram, we achieved *C*_*X*_ ≈ 0.2 fF and *R*_*X*_ ≈ 5 GΩ to yield a time constant of (*RC*)_*X*_ ≈ 10^–6^ s. Thus, when the frequency f of RF field is greater than 10 MHz, the smoothing condition time constant is much greater than the cycle time hold: (*RC*)_*X*_ ≈ 10^–6^ s >  > 10^–7^ s > 1/f.

#### Estimation of the ion flux

Table 1 presents the electrophysiological implications of our membrane model for a DC voltage of 1 µV. We estimated the total amount of ions *X* entering (or leaving) a single cell, which can be compared with the total number of ions in the cell Σ_*X*_. Based on Ohm’s law, the ion fluxes (*N*_*X*_) of the dominant potassium, sodium, and chloride ions through any open channel were estimated to be in the range of 10^3^ s^−1^. These values seem negligible compared to the complete ion content (Σ_*X*_) of the cell, which has billions of ions. However, when we considered an amplification factor of > 10^6^ (i.e., exposition time multiplied by the number of channels), the influxes of Na^+^ and Cl^−^ approached 50% of the entire cell content. The efflux of K^+^ relative to the intracellular inventory appeared less relevant. For the protons (H^+^), both the extra- and intracellular concentrations were 10^6^ times lower, which resulted in relatively similar influxes. For calcium ions (Ca^2+^), the extra-/intracellular concentration gradient was greater than 10^4^. Because of the low intracellular Ca^2+^ content, having only a few calcium channels open for a short time (e.g., tens of channels for a few minutes) would be sufficient to multiply the intracellular calcium concentration.

**Table 1:** Estimated resistance, ion current, ion flux, and resulting ion disequilibrium assuming Ohm’s law (for a DC voltage of 1 µV generated by E = 200 V/m) or maximal possible flux in file.

Ion *X*	Conductance *G*_*X*_ [pS]	Resistance *R*_*X*_ [GΩ]	Current *I*_*X*_ [fA] for 1 µV	Flux (out/in) *N*_*X*_ [s^−1^] for 1 µV	Ion content in cell Σ_*X*_	Relative ion loss/increase 10^6^ × *N*_*X*_/Σ_*X*_	Drift velocity *v*_*X*_ [μm/s]	Max flux (in file) *v*_*X*_/*d*_*X*_ [s^-1^]
K^+^	235	4.3	0.23	1.500 out	5 × 10^10^	0.03	15.2	38.000
Na^+^	144	6.9	0.14	900 in	2 × 10^9^	0.45	10.4	20.800
Cl^−^	174	5.7	0.17	1.100 in	2 × 10^9^	0.54	15.8	39.500
Ca^2+^	3	323	0.003	19 in	3 × 10^4^	> 100	12.3	30.800
H^+^	0.002	6 × 10^5^	2 × 10^–6^	10^–2^ in	3 × 10^4^	0.37	72.5	120.800

*G*_*X*_: Conductance of channel for ion *X*; *R*_*X*_: Resistance of channel for ion *X*; *I*_*X*_: Current of ion *X* through channel (according to Ohm’s law); *N*_*X*_: Ion flux (number of ions *X* transported through channel per second); Σ_*X*_: Total number of ions *X* in the cell; 10^6^ × *N*_*X*_/Σ_*X*_: Relative ion loss/increase per treatment using the amplification factor 10^6^ (see text); *v*_*X*_: Drift velocity of ion *X*; *d*_*X*_: hydration radius of ion *X* (Table 2)*; v*_*X*_/*d*_*X*_: maximum flux for moving in file.

The ratio of the drift velocity *v*_*X*_ caused by the additional DC voltage Δ*U*_*X*_ divided by the (hydrated) ion diameter *d*_*X*_ was used to estimate the maximum ion flux (last column of Table 1) under the assumption of a microscopic description (i.e., single-file ion movement). The yielded ion fluxes were 25–35 times higher for K^+^, Na^+^, and Cl^−^ and more than 10^3^–10^7^ times higher for Ca^2+^ and H^+^ (protons). Note that H^+^ demonstrates unique conduction in water with extreme mobility, which resulted in this substantially higher maximum flux^28^.

### Discussion

We verified the non-thermal effects of RF-EMF on colon cancer cell lines and established an electrophysiological model of the cell membrane and its ion channels to calculate the ion flux. This model can plausibly explain non-thermal effects in terms of electrochemical imbalances. Our findings do not question previous conclusions about the possible risks of RF-EMF and resultant safety levels^32^. We considered therapeutic levels of ≥ 25 W/kg and exposure times of 1 h (3,600 s), which are distinctly above the safety level (several watts per kilogram) and typical exposure time (seconds to minutes). In addition, normal tissues have less contact area between each cell and extracellular space, so they may be less sensitive than tumors.

However, the elucidated mechanisms may explain some unresolved observations in epidemiological studies^10,12^ and regarding EMF hypersensitivity^11,13^. More importantly, we may exploit these non-thermal effects for clinical purposes, particularly in oncology.

Note that we assumed an amplification factor of > 10^6^ for a single channel in conjunction with attainable therapeutic levels of 25 W/kg. Under these conditions, we estimated relevant displacements of K^+^ ions out of cells and Na^+^, Cl^−^, and Ca^2+^ ions into cells (Table 1) that can induce significant voltage shifts reaching the magnitude of the resting potential. Furthermore, the DC voltage of 1 µV generated by such therapeutic RF for a high number of channels may induce relevant ion fluxes for longer exposition times. However, the particular mechanisms that cause cell stress, which may result in apoptosis or cell death after a certain time, are still unexplored. Severe K^+^ efflux increases the extracellular potassium concentration and tends to depolarize the resting potential. In addition, intracellular loss of K^+^ may promote apoptosis^33^. An intense influx of NaCl may depolarize the cell membrane and provoke cell edema/swelling, which ultimately leads to cell death. The ion fluxes and ensuing electrochemical disturbances and redistributions can be increased by more than an order of magnitude if the ions move in single file as fast as physically possible. The last column of Table 1 presents the estimated maximum fluxes. Presently, it is not clear whether the macroscopic Ohm’s law or a microscopic description of the chemical reactions better reflects the actual course of events.

Table 1 indicates that a high influx of Ca^2+^ is the most likely to damage tumor cells. Even a single open calcium channel lets through nearly all of the Ca^2+^ content of a cell in approximately 1,500 s. RF is rather likely to induce a calcium overload of tumor cells and has already been documented^24^. Facilitated diffusion (i.e., single file) can distinctly increase the calcium inflow. While a moderate Ca^2+^ influx is indispensable to trigger numerous processes in the cell, Ca^2+^ overload is hazardous and particularly may increase the susceptibility to apoptotic cell death^33^.

The aberrant expression of ion channels is a well-known phenomenon in cancer^33^. Such altered channelomes have various functions in neoplastic transformation^34^. Onco-channels are often highly overexpressed at fivefold to over 100-fold^35^. Most of the literature on ion channels in cancer has pursued targeting these channels for treatment with approved drugs^36,37^. In the case of any RF exposition, overexpression of onco-channels may increase the abovementioned enhancement factor by even more than 10^6^. Therefore, localized heating of the tumor region, the specific intratumoral microenvironment, and altered channelomes of malignancies are three factors that may increase the therapeutic ratio.

In our study, we assumed continuously open ion channels, which limits the validity of our results. The probability that channels remain continuously open is quite variable. If 90–99% of the considered ion channels are closed, our numerical values would decrease by a factor of 10–100. Because we conservatively gauged amplification factors to be 10^6^, the true amplification factor may be higher and partially compensate for such diminution. In addition, channel opening may increase with a change in temperature of a few degrees Celsius, which is known to occur for Ca^2+^ channels. Further, other influences may open channels, particularly local shifts in the membrane potential.

In summary, RF-EMF has the potential to cause considerable ion fluxes that may adversely affect the proliferation and clonogenicity of cancer cells. Available preclinical and clinical data support the existence of non-thermal membrane/cellular injuries caused by RF-EMF. However, deciphering particular mechanisms would need further investigation. Our proposed theoretical framework paves the way for future experiments.

### Methods and materials

#### Cell cultivation

The two human colon cancer cell lines HT-29 and SW480 were used in the experiments. All cells were originally from the American Type Culture Collection and grown in a DMEM or RPMI 1,640 medium (Thermo Fisher Scientific, Waltham, MA) supplemented with 10% fetal bovine serum (Thermo Fisher Scientific). All cells were maintained at 37 °C in a humidified incubator with 5% CO_2_. All cells tested negative for mycoplasma, which was verified regularly with a MycoAlert Mycoplasma detection kit (Lonza, Basel, Switzerland). The cell lines were authenticated by short tandem repeat genotyping at the Leibniz-Institute DSMZ (Braunschweig, Germany). The short tandem repeat (STR) genotypes were consistent with published genotypes for these cell lines.

#### Application of hyperthermia

**Figure 2:**
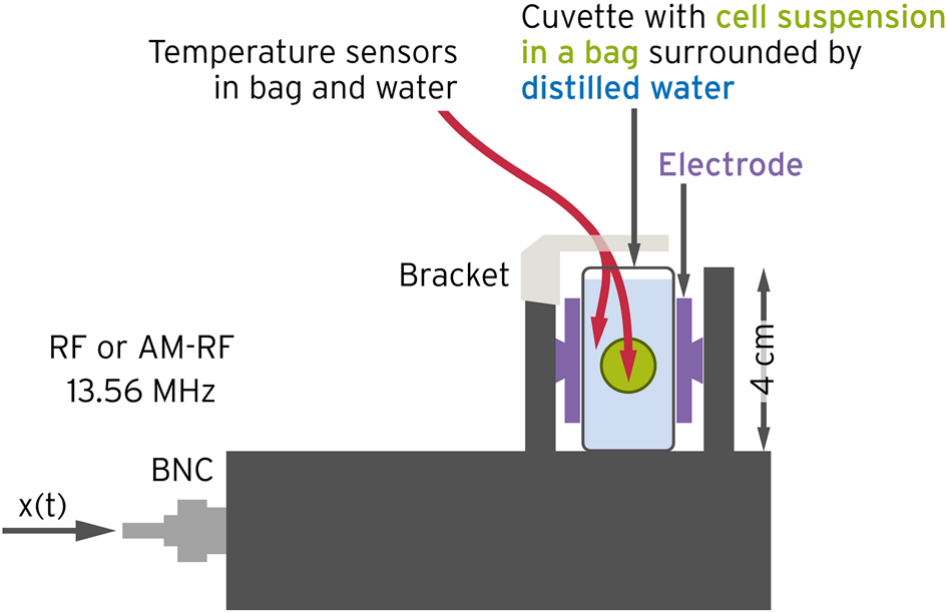
Experimental setup to apply RF-HT (LabEHY-200 in vitro applicator) to a cell suspension while adjusting the temperature (here 42 °C). The temperature was measured in the surrounding distilled water (blue) and at the center of the probe (green). We fed RF at 13.56 MHz to generate a nearly constant EMF between the electrodes (brown). Reflected power was minimized by automatic impedance matching.

Figure 2 depicts the experimental in vitro setup for RF-HT (LabEHY-200, Oncotherm Ltd, Troisdorf, Germany). An electrode chamber with dimensions of 2 cm × 2 cm × 4 cm was equipped with opposite copper electrodes with dimensions of 1.5 cm × 3 cm and filled with distilled water. A plastic bag with a cell suspension (1 × 10^6^ cells in 1.5 ml complete growth medium) was placed in the center. A pure sinusoidal RF of 13.56 MHz was fed to the applicator. A target temperature of 42 °C was set and maintained with temperature sensors in the center of the bag. The temperature sensors were used under sterile conditions and kept in 96% alcohol in a laminar flow cabinet. The heating process was controlled by a computer; a total power of 10–20 W and a typical temperature increase gradient of 0.7 ± 0.1 °C/min were used to raise the temperature from room temperature to the desired 42 °C. This corresponded to a SAR of ~ 40 W/kg in the probe^19^. After the target temperature was reached, the total power was automatically reduced to 5–10 W, and the steady-state period was maintained for 60 min. The equilibrium temperature in the surrounding water was slightly lower at around 40–41 °C, which resulted in a temperature drop from the center to the periphery of 0.2–0.5 °C^27^. Therefore, the mean temperature in the probe was slightly below 42 °C. The total energy during the heating and equilibrium phases slightly fluctuated around 50 kJ at ± 10%. For comparison, we also performed WB-HT; 1 × 10^6^ were cells placed in a tube with a preheated culture medium and incubated at 42 °C for 60 min.

#### Functional assays in vitro

To assess the effects of treatment on proliferation, cells were seeded after treatment into 96 transparent well plates at a density of 5 × 10^3^ cells per well. The cell growth was monitored in real-time by photo-documentation, and the phase-contrast confluence was automatically quantified by an IncuCyte cell monitoring device (Essen BioScience, Hertfordshire, United Kingdom) for at least 88 h.

To estimate the colony-initiating capacity of HT-treated cells, 5 × 10^2^ cells were placed in six well plates and cultivated for 7 days in 2 ml of complete medium. Colonies were subsequently fixed and stained with 1% formaldehyde and 0.1% crystal violet for 15 min followed by a thorough rinsing in tap water. After being dried, plates were photographed with a FluorChem Q Imager (ProteinSimple, San Jose, CA, USA). Colonies were counted and the area was measured with the Colony Counting routine in the AlphaView software (ProteinSimple).

We conducted all experiments in technical triplicate and at least biological triplicate.

#### Statistical analysis

The GraphPad PrismStatistical Analysis software (version 6.01) was used for all analyses. All reported effects were tested for statistical significance through one-way analysis of variance (ANOVA) and corrected for multiple comparison with Tukey’s range test.

#### Electrophysiological assumptions

**Figure 3:**
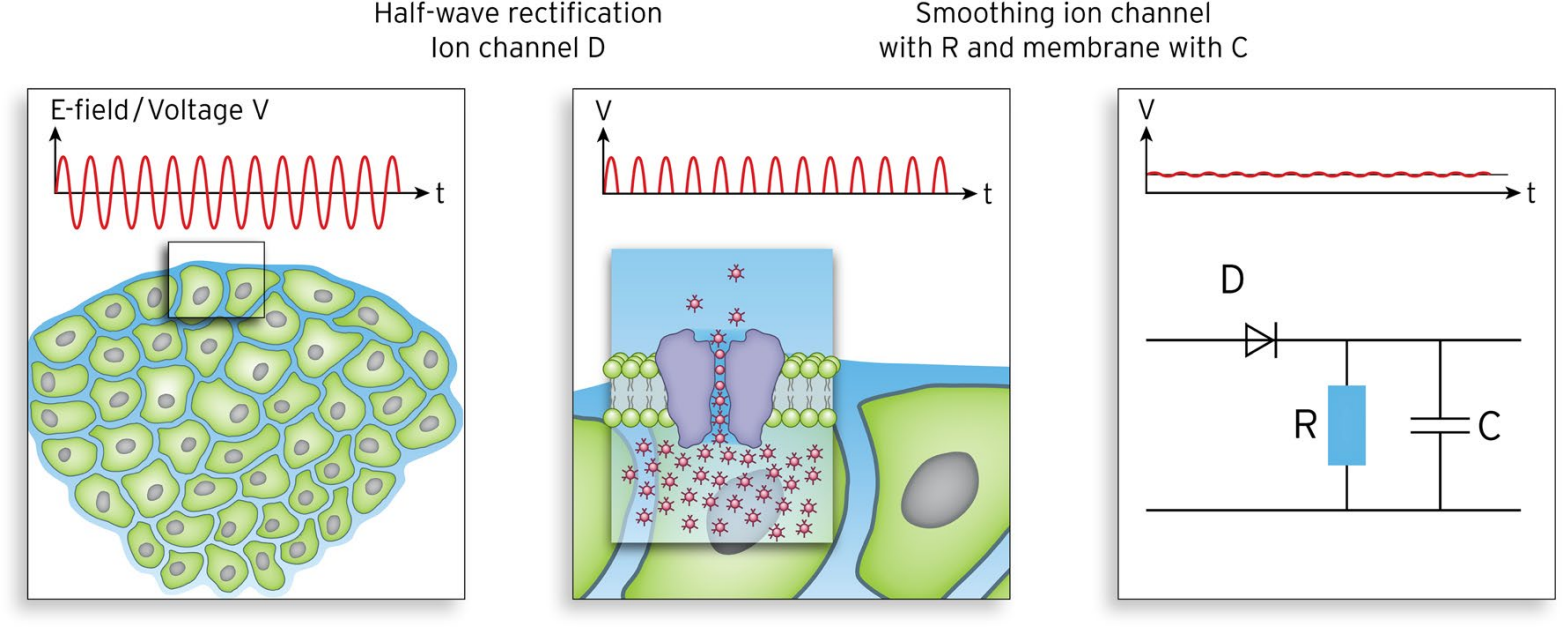
Left: Typical tumor environment characterized by isolated tumor cells surrounded by extracellular water. Middle: Simplified model of ion channels in the membrane (e.g., potassium) showing the internal pore, cavity, and selectivity filter. This model is an archetype for other ion channels. Right: Equivalent circuit diagram of this ion channel acting as a half-wave rectifier like diode D and a low-pass filter with resistance R of the ion channel and capacitance C of the adjacent membrane. The RF along the channel perpendicular to the membrane is transformed into a rippled DC voltage of around 1 µV for E = 200 V/m.

Cell membranes have a relatively constant specific capacitance (C_M_) of 1–3 µF/cm^2^^2,38^ but exhibit a wide range of specific membrane resistances (R_M_) from 10^8^ Ω cm^2^ to 5 Ω cm^2^; this strongly depends on the kind and number of ion channels (i.e., channelome) present in the membrane^7,40,41^, which vary to a large extent^28^. In conjunction with the intra-/extracellular ion concentrations, the channelome determines the negative resting membrane potential U_M_ described by the Goldman–Hodgkin–Katz (GHK) equation (Table 2), which can be -20 to -90 mV. According to the GHK equation for a single ion (i.e., Nernst equation), the equilibrium potential (U_X_) for every ion can strongly differ from U_M_ (Table 2)^38^. The mobility (u_X_) and hydration diameter (d_X_) are other notable features of every listed ion. The hydrated diameter consists of the ion itself and surrounding attached water molecules. Table 2 summarizes the parameters for relevant ions. Table 3 summarizes the physical laws and constants required to estimate the ion currents (or ion fluxes) through the ion channels in the membrane^28,29^. In particular, permanently open K^+^ channels^42^ are required to regulate U_M_ of every cell. Tumor cells may have a different channelome with a higher density^33,36^ of onco-channels, which results in different U_M_. The structure of the K^+^ channel KcsA has been deciphered by X-ray crystallography^42^ and was assumed to be a template for other ion channels such as Na^+^, Cl^−^, and Ca^2+^^35^. The crucial part of every channel is the pore, which acts as a selectivity filter for a particular ion^43^. We employed a model of a typical ion channel using available information^28,40,42^ to calculate ion fluxes (Fig. 3).

**Table 2:** Electrophysiological laws and variables to calculate the cell membrane potential and equilibrium potential of different ions.

Ion X	Extracellular concentration c_Xe_ [mmol/l]	Intracellular concentration c_Xi_ [mmol/l]	Ion potential (Nernst) U_x_ [mV]	Mobility u_X_ [10^–4^ cm/Vs]	Hydration diameter d_X_ [nm]
K^+^	4.5	160	− 95	7.62	0.25
Na^+^	144	7	+ 80	5.19	0.35
Cl^−^	114	7	− 80	7.92	0.20
Ca^2+^	1.3	< 10^–4^	> 125	6.17	0.30
H^+^					
Normal tissue	0.5 × 10^–4^ (pH = 7.4)	10^–4^ (pH = 7.1)	− 24	36.25	0.60
Tumor	2.5 × 10^–4^ (pH = 6.1)	10^–4^ (pH = 7.1)	+ 24		
Fundamental (Goldman–Hodgkin–Katz (GHK) equation		${U}_{M}\left[\text{mV}\right]=61\times \text{lg}\frac{{P}_{K}{\left[K\right]}_{e}+{P}_{Na}{\left[Na\right]}_{e}+{P}_{Cl}{[Cl]}_{i}}{{P}_{K}{\left[K\right]}_{i}+{P}_{Na}{\left[Na\right]}_{i}+{P}_{Cl}{[Cl]}_{e}}$			
Nernst equation (as outlier for single ion X)		${U}_{X}\left[\text{mV}\right]=-(61/{z}_{X})\text{lg}({c}_{Xi}/{c}_{Xe})$			

The membrane potential U_M_ depends on the ion concentration c_X_ and permeability P_X_ and describes the penetrability of the membrane for ion X. P_X_ can be estimated from the ion channel for ion X either by the classical macroscopic theory (diffusion equation, Ohm’s law) or by detailed microscopic considerations using rate equations of the underlying chemical reactions. The mobility u_X_ and hydration diameter d_X_ are needed to estimate the ion flux. P_X_ is determined by the channelome.

**Table 3:** List of physical laws and constants used to estimate current or flux of ion X.

Parameter/constant	Equation	Number
E-field (e.g., 200 V/m for 25 W/kg)	$E[\text{V}/\text{m}]=45\times {(SAR[\text{W}/\text{kg}]/\sigma [\text{S}/\text{m}])}^{1/2}$	(1)
Membrane capacitance	${C_M}[\upmu {\text{F}}/{\text{c}}{{\text{m}}^2}] = {\varepsilon _{rM}}{\varepsilon _0}/{d_M} \cong 1\;\left( {{\text{for}}\;{\varepsilon _{rM}} = 5,\;d = 5\;{\text{nm}}} \right)$	(2)
Conductance	${G}_{X}\left[S\right]=\frac{1}{{R}_{X}\left[\Omega \right]}={z}_{X}\times F{u}_{X}\times {c}_{X}\times ({a}^{2}/L)$	(3)
Voltage induced by E across membrane ΔU_M_	$\left[V\right]={10}^{-8}\times {E}_{\perp }[\text{V}/\text{m}]$	(4)
Ohm’s law	${I}_{X}[A\equiv C/s]=\Delta {U}_{m}[\text{V}]\times {G}_{X}[S]$	(5)
For U_M_ = 100 mV	${U}_{M}=100\; \text{mV} \;\text{at} \; {C}_{M}=2\frac{\upmu \text{F}}{\text{cm}^{2}} \text{: 2 }\frac{{10}^{-7}C}{{\text{cm}}^{2}}\triangleq {10}^{4} \text{ ions}/{\upmu \text{m}}^{2}$	(6)
Drift velocity	${v_X}[\upmu {\text{m}}/{\text{s}}] = E[{\text{V}}/{\text{cm}}]{u_X}[{10^{ - 4}}\;{\text{c}}{{\text{m}}^2}/{\text{Vs}}]$	(7)
Elementary charge	${q}_{e}=1.60\times {10}^{-19} \;\text{C}$	(8)
Avogadro constant	${N}_{A}=6\times {10}^{23}\;\text{molecules}/\text{mol}$	(9)
Faraday constant	$F={N}_{A}\times {q}_{e}=9.65\times {10}^{4} \;\text{C}/\text{mol}$	(10)
Dielectric field constant (free space)	${\varepsilon }_{0}=8.85\times {10}^{-12} \;\text{C}/\text{V}/\text{m}$	(11)

For the conductance G_X_ of a channel, we inserted a mean edge length a = 1 nm and membrane thickness L = 5 nm. The conductivity in the intra-/extracellular water was σ = 1.2 S/m (Fig. 3). The charge number was z_X_ = 1 or 2 (for Ca^2+^). For the mobility u_X_, see Table 2.

#### Electrical model of cell membrane and ion channels

To establish the model, we made the following assumptions. A given RF (e.g., 13.56 MHz) has an EMF amplitude E [V/m] derived from SAR [W/kg] (Eq. 1). A SAR of 25 W/kg resulting in E ≈ 200 V/m was predefined to agree with the mean SAR for the equilibrium period of our experiments and to be feasible in clinical practice. An open ion channel like that shown in Fig. 3 strongly prefers the current direction to bring U_M_ of the membrane closer to the equilibrium potential of the related ion. In the case of potassium, an outflux is preferred, while an influx is preferred for sodium and calcium.

#### Estimation of ion fluxes

The resulting currents through open channels can be estimated according to Ohm’s law (Eq. 5). In addition, the ion number at the membrane surface area around any channel to generate a significant shift in the membrane voltage can be calculated (Eq. 6). A small number of several thousand ions per channel (here, 10,000 ions) appears to be sufficient to cause a significant voltage change of 100 mV, which would result in complete depolarization. However, the time dependence of these processes and the dynamics of ion attenuation at the membrane are poorly understood.

To estimate the total ion flux into or out of a single cell, we assumed a mean cross-section of 1 nm × 1 nm for a channel with a length of 5 nm and determined the electrical parameters G_X_ and R_X_, as suggested by others^28^. Then, the current I_X_ and number of ions N_X_ crossing an open channel per second were calculated. We further assumed a mean density of 10 channels/µm^2^ (i.e., 1,000 channels in the exposed membrane area of 10 μm × 10 μm)^39^ and an exposition time of > 3,000 s (60 min) for a typical modulated electro-hyperthermia (mEHT) session. By estimating the total ion disequilibrium (loss or increase) for a single cell during long-term exposition (> 1,000 s) from the ion flux (per second) of a single channel, we achieved an amplification factor of > 10^6^.

For the equivalent circuit diagram shown in Fig. 3, the capacitance C_X_ of any channel embedded in the membrane with ~ 2 µF/cm^2^ was calculated for a circle with a radius of 50 nm around the channel. Then, R_X_ and C_X_ were used to characterize the electrical behavior of the circuitry^44^. To estimate the maximum facilitated ion flux beyond Ohm’s law, we also utilized the drift velocity (v_X_) of ion X through channel X that was caused by the generated DC voltage. Equations 4 and 7 were used to estimate v_X_. This was done because the transition-state theory, which describes discrete ion movement (hopping) driven by chemical reactions^45,46^, may be more applicable than Ohm’s law for calculations at a microscopic scale^29^. Fast chemical reactions may facilitate ion flux beyond that predicted by Ohm’s law and enable a maximum diffusion rate of 10^8^ s^−1^. For example, the conduction of an ion stalled in the pore is accelerated by repulsive forces when a second ion enters^42^, as indicated in Fig. 3. In other words, at maximum speed a continuous chain of ions can move in single file through a pore.
